# Shaking B Mediates Synaptic Coupling between Auditory Sensory Neurons and the Giant Fiber of *Drosophila melanogaster*

**DOI:** 10.1371/journal.pone.0152211

**Published:** 2016-04-04

**Authors:** Adeline P. Pézier, Sami H. Jezzini, Jonathan P. Bacon, Jonathan M. Blagburn

**Affiliations:** 1 Institute of Neurobiology, University of Puerto Rico Medical Sciences Campus, San Juan, Puerto Rico, United States of America; 2 School of Life Sciences, University of Sussex, Brighton, United Kingdom; Universitaet Regensburg, GERMANY

## Abstract

The Johnston’s Organ neurons (JONs) form chemical and electrical synapses onto the giant fiber neuron (GF), as part of the neuronal circuit that mediates the GF escape response in *Drosophila melanogaster*. The purpose of this study was to identify which of the 8 *Drosophila* innexins (invertebrate gap junction proteins) mediates the electrical connection at this synapse. The GF is known to express Shaking B (ShakB), specifically the ShakB(N+16) isoform only, at its output synapses in the thorax. The *shakB*^*2*^ mutation disrupts these GF outputs and also abolishes JON-GF synaptic transmission. However, the identity of the innexin that forms the presynaptic hemichannels in the JONs remains unknown. We used electrophysiology, immunocytochemistry and dye injection, along with presynaptically-driven RNA interference, to investigate this question. The amplitude of the compound action potential recorded in response to sound from the base of the antenna (sound-evoked potential, or SEP) was reduced by RNAi of the innexins Ogre, Inx3, Inx6 and, to a lesser extent Inx2, suggesting that they could be required in JONs for proper development, excitability, or synchronization of action potentials. The strength of the JON-GF connection itself was reduced to background levels only by RNAi of *shakB*, not of the other seven innexins. ShakB knockdown prevented Neurobiotin coupling between GF and JONs and removed the plaques of ShakB protein immunoreactivity that are present at the region of contact. Specific *shakB* RNAi lines that are predicted to target the ShakB(L) or ShakB(N) isoforms alone did not reduce the synaptic strength, implying that it is ShakB(N+16) that is required in the presynaptic neurons. Overexpression of ShakB(N+16) in JONs caused the formation of ectopic dye coupling, whereas ShakB(N) prevented it altogether, supporting this conclusion and also suggesting that gap junction proteins may have an instructive role in synaptic target choice.

## Introduction

**Fig 1:**
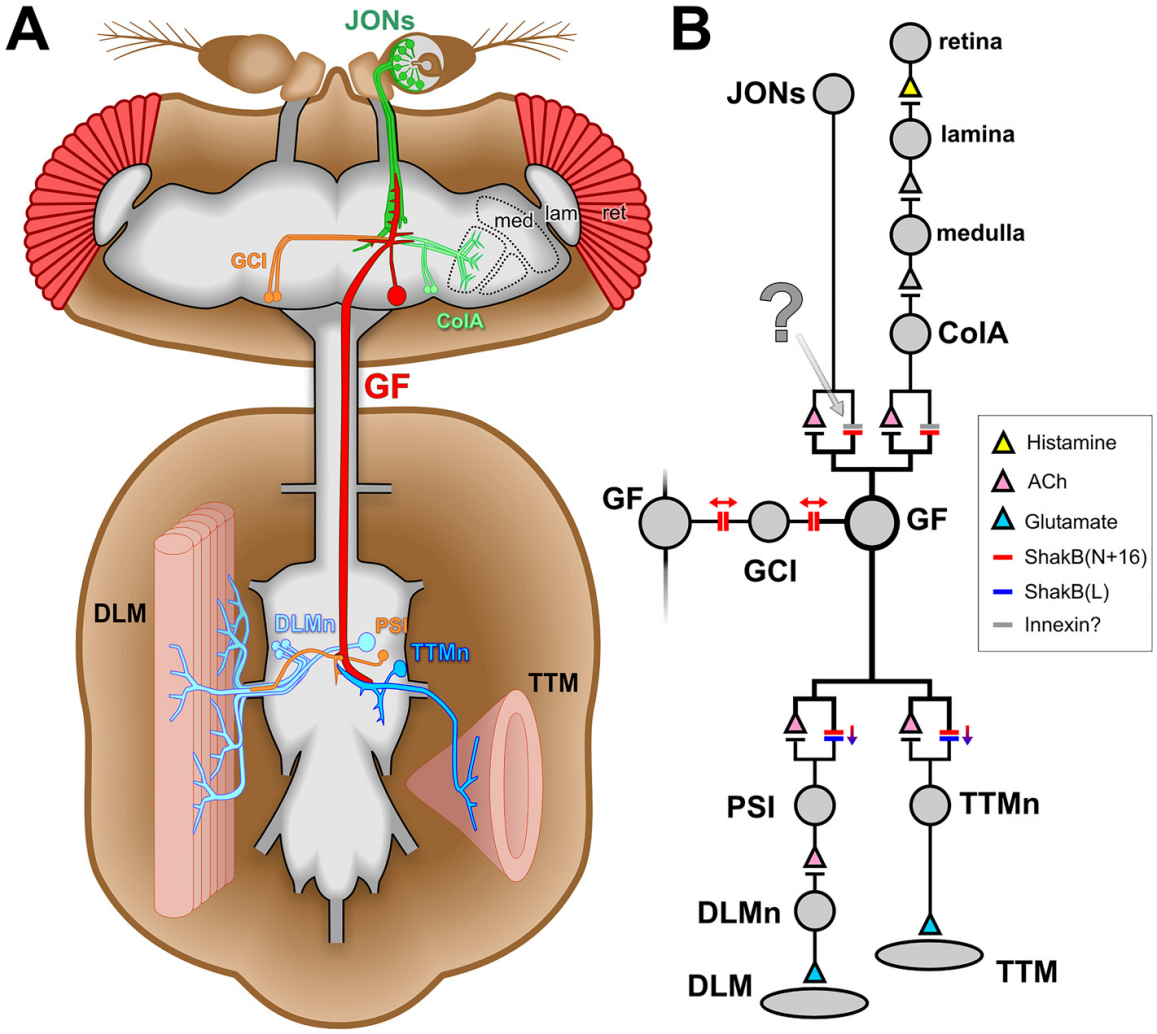
The *Drosophila* giant fiber escape circuit. (A) Diagram of the anatomy of one half of the system, it being duplicated contralaterally. In the brain, the giant fiber (GF) receives synaptic inputs onto its dendritic branches from the mechanosensory Johnston’s organ neurons (JONs) and from polysynaptic visual pathways (ret: retina, lam: lamina, med: medulla, ColA: lobular ColA interneurons). It also forms synaptic connections with the giant commissural interneurons (GCI). The GF axon descends to the thoracic ganglion where it forms electrical and chemical synapses with the tergotrochanteral motorneuron (TTMn) of the cylindrical tergotrochanteral (jump) muscle (TTM) and the peripherally-synapsing interneuron (PSI) which innervates the dorsal longitudinal motorneurons (DLMn) of the dorsolongitudinal flight muscles (DLM). (B) Wiring diagram of the circuit. Chemical synapses are denoted by triangles, colored to represent the transmitter where it is known. Electrical synapses are denoted by double bars, colored to represent which innexin is involved, with arrows indicating putative rectifying or non-rectifying junctions. The synapse investigated in the current study is indicated with a question mark. Evidence compiled from several publications [5,7,9–11].

Electrical synapses are a typical feature of escape circuits in vertebrates and invertebrates [1]. The gap junctions that comprise them are formed from connexin proteins in vertebrates and the functionally analogous innexins in invertebrates [2]. So far, 8 genes for innexins have been identified in the genome of *Drosophila melanogaster* [3]. One of the most thoroughly studied, Shaking-B (ShakB), is a critical component of the giant fiber (GF) portion of the escape circuitry [4–6] (Fig 1). ShakB-containing electrical synapses are present, along with cholinergic chemical synapses [7], at contacts made by the GF with two of its output neurons, the peripherally-synapsing interneuron (PSI) and the tergotrochanteral motor neuron (TTMn) [8] (Fig 1).

There is also evidence that ShakB is required for excitatory inputs onto the front end of the GF: the *shakB*^*2*^ mutation disrupts visual circuitry [12], and abolishes synaptic currents from auditory neurons in the Johnston’s Organ (JO) of the antenna [13]. In a recent study, we also found evidence that, despite the presence of chemical contacts, transmission of the response to sound at the JO neuron (JON)—to—GF synapses is indeed primarily electrical [14]. In that same study we observed that the tracer Neurobiotin (NB) passes retrogradely from GF to a subset of JONs, but that the slightly larger molecule Lucifer Yellow (LY) does not. In contrast, both tracers readily diffuse from the GF into the giant commissural interneurons (GCIs), via gap junctions that may synchronize activity in the two GFs on either side of the brain [4].

The *shakB* locus codes for a nested set of at least 5 transcripts [15–17] (although there are 8 potential ones listed in Flybase), which are translated into three distinct proteins: Shaking-B(Lethal), Shaking-B(Neural), and Shaking-B(N+16) [5]. The *shakB*^*2*^ mutation disrupts the latter two only. Only the *shakB(n+16)* transcript has been identified in the GF [5]. The GF-TTMn gap junctions are rectifying, and are heterotypic, being composed of ShakB(N+16) on the presynaptic side and ShakB(L) on the postsynaptic [5]. Conversely, those between the GF and the GCIs are thought to be homotypic and non-rectifying, with ShakB(N+16) on both sides [5] (Fig 1B).

In view of our aforementioned observation of the different dye-coupling properties of the JON-GF and GCI-GF synapses [14], we surmised that perhaps the JON-GF synapses would therefore not be homotypic, and so be composed of ShakB(N+16) in the GF dendrites coupled with an as-yet-unidentified innexin presynaptically. In the present study we test the requirement for all 8 different innexins at this synapse using RNA interference (RNAi) knockdown within the presynaptic JONs. We find, in fact, that it is only ShakB that is required presynaptically for both dye coupling and electrical transmission. Intriguingly, we also find that expression of ShakB(N+16) in a subset of JONs that do not normally express ShakB and do not couple to the GF leads to the formation of inappropriate synaptic connections with it, while presynaptic overexpression of ShakB(N) appears to inhibit gap junction formation altogether.

## Materials and Methods

### Flies

*Drosophila melanogaster* flies of the following genotypes were obtained from the Bloomington Stock Center: *UAS-ogre-RNAi* (44048: VALIUM20), *UAS-Inx2-RNAi* (42645: VALIUM20), *UAS-Inx3-RNAi* (30501: VALIUM10), *UAS-zpg-RNAi* (27674: VALIUM10), *UAS-Inx5-RNAi* (28042: VALIUM10), *UAS-Inx6-RNAi* (44663: VALIUM20), *UAS-Inx7-RNAi* (26297: VALIUM10), *UAS-shakB{JF02603}-RNAi* (27291: VALIUM10, affects ShakB(N) isoform), *UAS-shakB{JF02604}-RNAi* (27292: VALIUM10, affects ShakB(L) isoform), *UAS-shakB{HMC04895}-RNAi* (57706: VALIUM20, affects all isoforms), *UAS-mCD8*::*GFP* (5130 or 5137), *UAS-Dcr-2* (24650 or 24651), *JO15-GAL4* (6753), *GMR79H05-GAL4* (40054). *UAS-shakB{GD12666}-RNAi* (v26801, affects all isoforms) was obtained from the Vienna Drosophila RNAi Center [18]. Other lines used were *UAS-shakB(N)* and *UAS-shakB(N+16)* {Pauline Phelan [5]}, *shakB*^*2*^/FM7c {Tanja Godenschwege [19]}, *peb-GAL4* and *peb-GAL4*, *mCD8*::*GFP* on the X chromosome {Liqun Luo [20]}. A GFP-tagged chromosome 2 balancer containing *CyO*, *Kr-Gal4*, *UAS-GFP* (denoted *CyO-GFP* below) was obtained from Bruno Marie [21]. The following fly lines were constructed in the laboratory:

*peb-GAL4;+;UAS-Dcr-2*/*TM6B*, *Tb*^*1*^,

*peb-GAL4*, *mCD8*::*GFP;UAS-Dcr-2*/*CyO-GFP*,

*UAS-mCD8*::*GFP/ CyO-GFP;JO15-GAL4/TM6B*, *Tb*^*1*^,

*UAS-Dcr-2/ CyO-GFP; JO15-GAL4*/*TM6B*, *Tb*^*1*^,

*UAS-mCD8*::*GFP/ CyO-GFP;GMR79H05-GAL4/TM6B*, *Tb*^*1*^.

*GAL4* lines were crossed with the respective *UAS* lines and the F1 used for experiments. Flies were reared on cornmeal media and raised at 25°C and 60% relative humidity. In some cases, to increase *GAL4* activity, flies were transferred to 30°C or, to decrease it, to 20°C [22]. Adults from 3–10 days old were used for experiments. For electrophysiology, mainly females were used, except for *R79H05-GAL4>UAS-shakB-RNAi* animals, where both sexes were used due to the low yield. For dye-coupling, both sexes were used. The genotypes, rearing temperature, and survival rates for all animals tested are listed in S1 Table.

### Dye coupling

Dissection and dye injection were performed as previously described [14]. Briefly, animals were dissected so as to expose the cervical connective and reveal the GF axons. One of the GF axons was impaled with a sharp glass microelectrode and injected with a mixture of 3% Neurobiotin Tracer (NB) (Vector Labs, SP-1120) and 2% Lucifer Yellow CH, lithium salt (LY) (Molecular Probes, OR, USA, L-453) diluted in water. Injection electrodes were backfilled with 0.5 μl of dye mixture followed by 150 mM potassium chloride and had resistances of 45–60 MΩ resistance. The dye mixture was iontophoretically injected for up to 20 minutes using a continuous train of alternating 1 second square pulses of positive and negative current of ± 1–2 nA generated by a Master 8 stimulus generator (A.M.P.I., Israel) and delivered through an AxoClamp 2B amplifier (Molecular Devices LLC, CA, USA).

### Immunohistochemistry

The nervous systems from dye-injected flies were fixed in 4% paraformaldehyde in phosphate-buffered saline (PBS) for 45 minutes at 4°C, rinsed in several changes of PBS and further dissected to remove brain and ventral nerve cord. To process other flies, nervous systems were dissected in PBS, fixed in 4% paraformaldehyde in PBS for 30 minutes at room temperature and rinsed in several changes of PBS. After removal, nervous systems were processed for antibody labeling, and cleared and mounted as previously described [14]. Rabbit polyclonal anti-ShakB [5] was used at a dilution of 1/500. Anti-Inx2 antibody was a gift from Pauline Phelan and was used at a dilution of 1/500 [23]. Rabbit polyclonal anti-Ogre was a gift from Andrea Brand and used at 1/200 [24]. Rabbit polyclonal anti-Inx7 was a gift from Michael Hoch and was used at 1/1000 [25]. Rabbit polyclonal anti-Lucifer Yellow (Molecular Probes, cat. A5750) was used at 1/2000. Goat anti-rabbit secondary antibody labeled with Alexa-555 (Molecular Probes, A21428), or donkey anti-rabbit secondary antibody labeled with Alexa-568 (Molecular Probes, A10042), was applied at 1/500. Streptavidin-conjugated Pacific Blue (Molecular Probes S11222) was added at 1/2000 during incubation in primary and secondary antibody. Preparations were examined using either a Zeiss Pascal or Nikon Eclipse T1 A1r laser scanning confocal microscope and images were acquired at 8 bit resolution.

### Electrophysiology

Flies were briefly chilled to immobilize them before mounting on a slide with dental wax. Recordings of the sound-evoked potential (SEP) from the antennal nerve, and sound delivery were performed as previously described [26]. As in other studies [14,26–28], SEPs were recorded extracellularly from near the antennal nerve, with an electrode inserted shallowly into the ventro-medial aspect of the antennal scape (first segment). The indifferent electrode was also inserted in a defined position between the anterior and middle orbital bristles. For simultaneous recording from the antennal nerve and GF system, recordings were performed as described [14]. The dorsal longitudinal flight muscle (DLM) and tergotrochanteral jump muscle (TTM) of the opposite side were recorded using glass electrodes filled with 50:50 mix of 1M KCl and 1M K acetate. Signals from each muscle were amplified x10, one with a Neuroprobe 1600 amplifier (A-M Systems, WA USA), the other one with a Intra 767 Electrometer (WPI Inc., FL USA). Signals were amplified another x10 and filtered at 30 kHz with a Brownlee Precision 210A amplifier, digitized with a Digidata 1320A, acquired and sampled at 50 kHz with pClamp. A tungsten electrode was placed into the abdomen to ground the preparation. In this assay we exclusively use the long-latency (3–5 ms) muscle response, obtained when a low intensity voltage is applied across the eyes [29] and attributable to the activation of the GF through the polysynaptic visual pathway. Voltage stimuli (approx. 3V) were applied with a tungsten electrode inserted shallowly into each eye, connected to a S48 stimulator and SIU5 Isolation Unit (Grass Technologies, RI USA), and were of 0.6 ms duration and given at 0.3 Hz to avoid habituation [30]. The voltage stimulus intensity necessary to reach the threshold for eliciting long-latency responses was determined and adjusted for each experiment so as to be just sub-threshold, so that muscle spikes, indicative of a GF action potential, occurred in only ≤ 1 in 10 trials in the absence of sound, presumably due to stochastic fluctuations in the amplitude of the lobula synaptic inputs. If these criteria for adjusting the subthreshold stimulus voltage were consistently followed, the results obtained fell within the same range across preparations.

The auditory stimulus consisted of 200-Hz sine-waves of 100 ms duration, since these elicit strong, well-defined responses in the JONs, and in the GF [26]. Auditory stimuli elicit action potentials in a subpopulation of JONs that can be recorded extracellularly as trains of SEPs in the antennal nerve. Each of these SEPs is followed one-to-one by an excitatory postsynaptic current in the GF at 0.4 ms latency [13]. As in our previous study, we focused only on the first two SEPs in the train [14]. For each of the two presynaptic SEPs investigated, the delay of the voltage stimulus across the eyes was adjusted so that the GF would receive the resultant putative lobula input and the antennal input in synchrony. The experiment then consisted of testing a series of sound levels varying from 78 to 98 dB (SPL) for the two SEPs with, for each sound level, 10 trials in presence of sound alternating with 10 trials in absence of sound, and a total of 50–100 trials per experimental animal. The timing of the sub-threshold voltage stimulus relative to the SEPs was critical. It was found empirically that optimal responses were achieved if the voltage stimulus onset preceded the peak of the SEP of interest by about 3.5 ms, so that the small transient of unknown origin that invariably precedes the TTM action potential (which probably indicates the TTMn spike) coincided with the end of the SEP. SEP amplitudes were measured using Clampfit (Molecular Devices) and binned for each SEP investigated for each animal. The probability of the GF spiking was calculated as the number of trials with GF firing divided by the total number of trials for that amplitude bin. The probability of the GF spiking per amplitude bin was then averaged across animals of the same genotype. The GF was scored as spiking when both the TTM and the DLM fired with the appropriate latency.

For assessing the GF output synapses, we used the short-latency (about 1 ms) muscle response, obtained when a high intensity voltage (>15V) is applied across the eyes and attributable to the activation of the GF directly (by-passing the polysynaptic visual pathway) [31]. Only the stimulus across the eyes was used and no sound stimulation was applied. The stimulus duration was 0.03 ms. The voltage stimulus intensity necessary to reach the threshold for eliciting short-latency responses was determined and adjusted for the experiment so as to be maintained above threshold. To determine the TTM and DLM latencies, 10 single stimuli given at an interval of 5s were recorded and the average latencies calculated. To determine the following frequency at 200Hz and 50Hz, 10 trains of 10 stimuli at 200Hz and 50Hz giving an interval of 2s between trains were recorded and the percentage of the total responses calculated. Experiments were performed at a room temperature of about 19°C. We consistently obtain TTM and DLM latencies around 1.1 ms and 1.5 ms respectively across control fly lines. Those latencies are longer than typically reported but consistent with the room temperature at which the experiments were carried out [30].

### Figure Preparation

Confocal image stacks were imported into ImageJ (Wayne Rasband, NIH), where they were adjusted for optimal contrast. Maximum intensity z-series projections created in ImageJ were imported into Adobe Photoshop for construction of figures. For clarity, prior to making some projections, non-JO neuronal GFP fluorescence was partially digitally masked from the stack. The Fluorender program was used in the production of some of the neural renderings [32], and Blender (blender.org) was used for the construction of some of the 3D diagrams. Final graphics for all figures were composed and labeled using CorelDraw.

### Statistics

**Fig 2:**
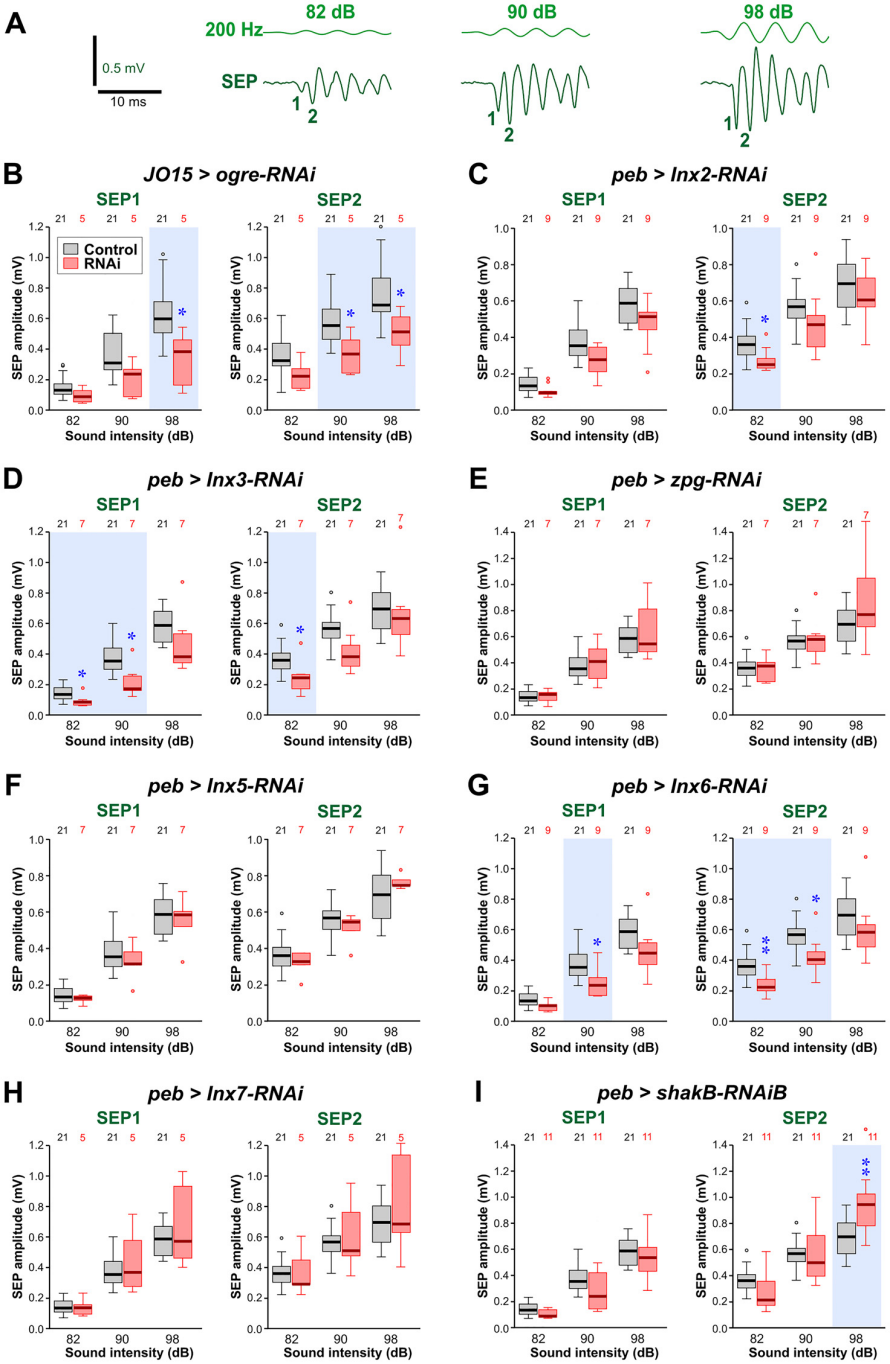
Effects of Innexin knockdown on the amplitudes of sound-evoked potentials. (A) Sample traces of sound-evoked potentials (SEPs) recorded from the base of the antenna, in response to 200 Hz sound pulses. As the sound level is increased (dB), the SEP amplitude also increases. The first two SEPs in the train are numbered 1 and 2. (B-I) Amplitudes of SEP1 and SEP2 in animals with RNA interference constructs targeting different innexins, compared to controls (*JO15-GAL4/+* in B and *peb-GAL4 > Dcr-2* in C-I). Paired t-tests or Mann-Whitney tests (with a Bonferroni correction for 3 comparisons) were used to determine significant differences from control. These are indicated with blue asterisks and a light blue background tint. (B) *JO15-GAL4* driving *ogre-RNAi* (short hairpin, so Dcr-2 not required). The *JO15-GAL4* driver was used instead of *peb-GAL4*, which proved lethal. Both SEPs were reduced at higher sound intensities. (C) *peb-GAL4* driving *Inx2-RNAi (*short hairpin). A reduction was seen in SEP2 at low intensity. (D) *peb-GAL4* driving *Inx3-RNAi* and *UAS-Dcr-2*. Both SEPs were reduced for low or medium sounds. (E) *peb-GAL4* driving RNAi of *zpg (Inx4*) and *UAS-Dcr-2*. No changes in SEPs were seen. (F) *peb-GAL4* driving *Inx5-RNAi* and *UAS-Dcr-2*. No changes in SEPs were seen. (G) *peb-GAL4* driving *Inx6-RNAi (*short hairpin). Reduction was seen in both SEPs for low and medium sounds. (H) *peb-GAL4* driving *Inx7-RNAi* and *UAS-Dcr-2*. No changes in SEPs were seen. (I) *peb-GAL4* driving *shakB-RNAi* (Bloomington short hairpin). A small increase was seen in SEP2 for loud sounds. For full genotypes see S1 Table.

**Fig 3:**
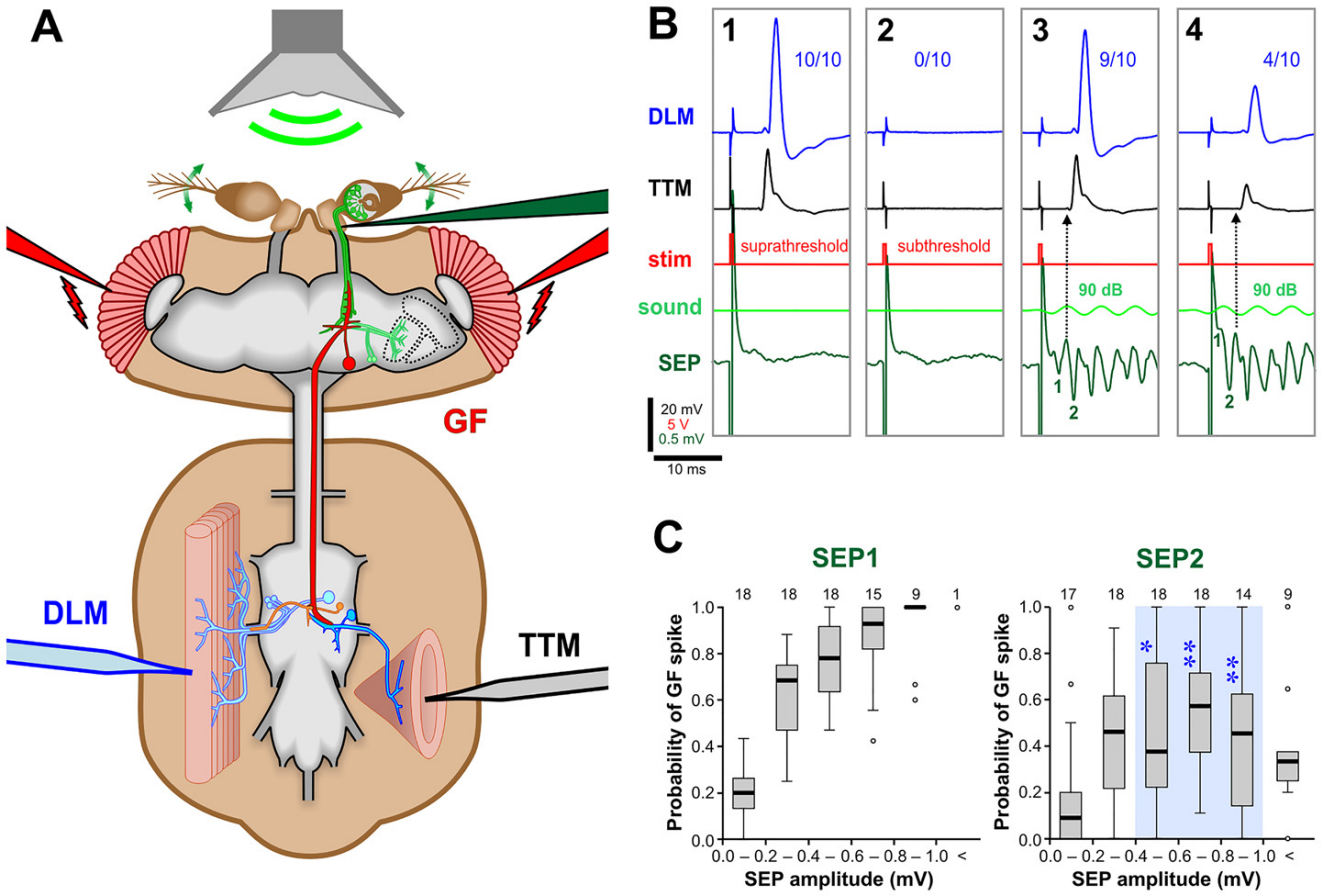
The strength of the synapse between JONs and the GF can be monitored through indirect recording of the GF system. (A) Electrophysiological recording set up. A low-voltage stimulus across the eyes (red electrodes) was used to stimulate the visual pathway. A 200-Hz sine-wave sound stimulus (green) delivered with a speaker was used to stimulate the JONs. Sound-evoked potentials (SEPs) were recorded from the antennal nerve with an electrode (dark green) inserted at the base of the antenna. The output of the GF pathway was recorded from the TTM (black electrode) and the DLM (blue electrode). (B1-4) Averages of 10 recording traces. (B1) A suprathreshold voltage stimulus depolarizes the GF above threshold, resulting in action potentials in both DLM and TTM in all 10 trials (10/10). (B2) For subsequent experiments, the voltage stimulus was adjusted so that the GF was just under its threshold for action potentials (subthreshold). Note the large stimulus artifacts in the antennal nerve traces. (B3) A 90 dB sound stimulus summates with the subthreshold voltage stimulus to activate the GF. The GF fires an action potential in 9 of 10 traces, making the averaged amplitudes of the TTM and DLM spikes slightly lower than those in B1. The delay of the voltage stimulus was adjusted in order to measure the GF response to SEP1. (B4) The stimulus delay was increased to measure the response to SEP2. The GF response occurs in only 4 of 10 traces, despite the larger SEP2 amplitude, making the averaged amplitude even smaller. SEP1 is obscured by the stimulus artefact. (C) Box-whisker plots of the GF’s response to SEPs of increasing amplitudes (a measure of the strength of the synapse between JONs and GF), SEPs of different amplitudes being achieved by altering the sound intensity. An increase in amplitude of SEP1 gives an increase in GF spike probability. SEP2 has significantly less excitatory effect on GF, particularly at large amplitudes. The flies are genotype *peb-GAL4; UAS-Dcr-2*. Numbers of animals are indicated along the top. Statistically significant differences between SEP2 versus SEP1 (Mann-Whitney test, Bonferroni p-correction for 5 comparisons) are indicated with blue asterisks and a light blue background tint. For full genotype, see S1 Table.

**Fig 4:**
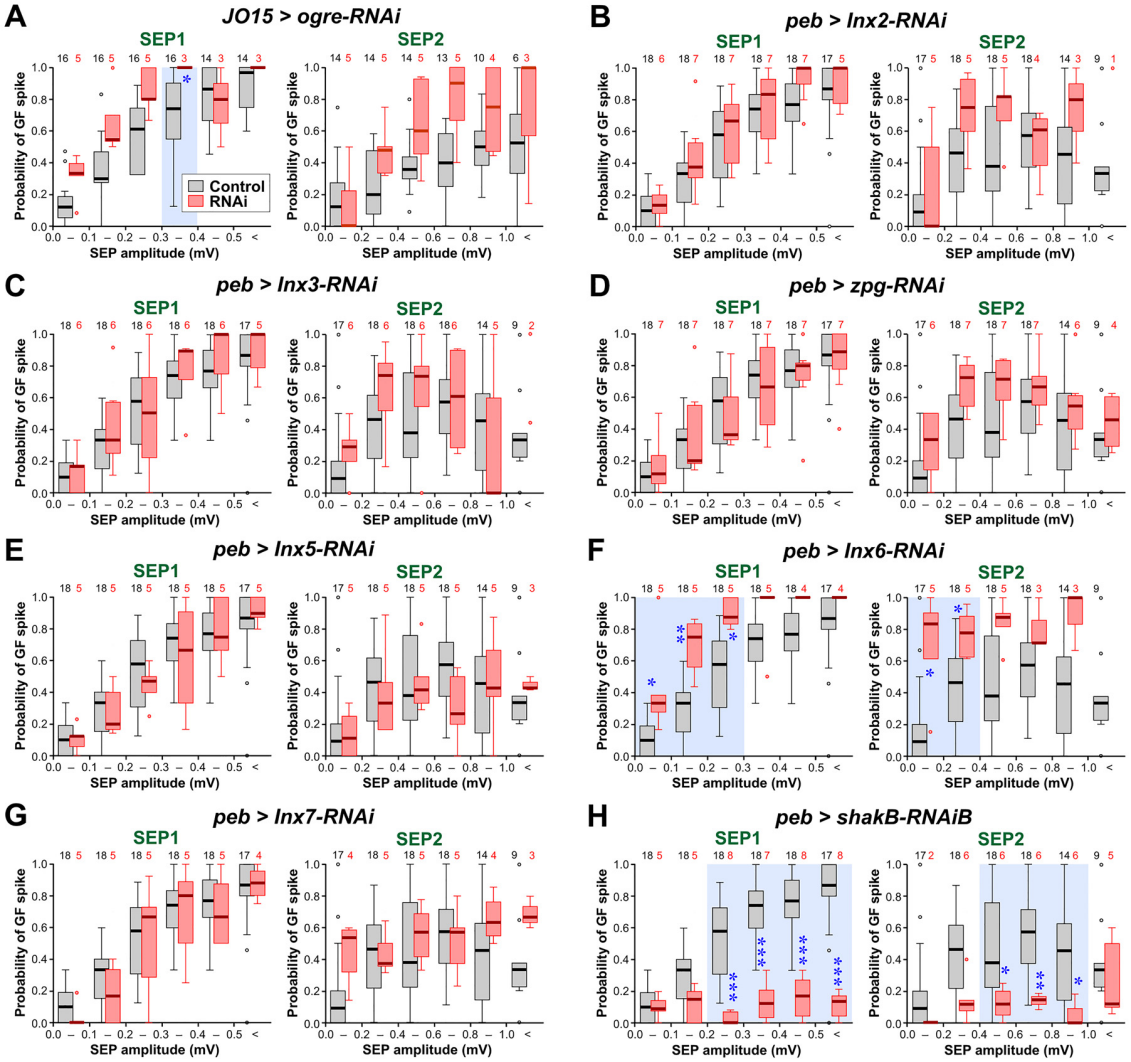
Effects of Innexin knockdown with RNAi on the strength of the JON-GF connection. Box-whisker plots of the probability of GF spiking in response to SEP1 and SEP2. Controls are shown in gray and correspond to *JO15-GAL4/+* in A and *peb-GAL4 > Dcr-2* in B-H. Experimental RNAi data are shown in pink. Ogre-RNAi (A) is driven by *JO15-GAL4*; all the other innexin RNAis are driven by *peb-GAL4* with or without *UAS-Dcr-2* depending on the RNAi construct. The numbers of animals are indicated along the top. Statistically significant differences between RNAi versus control (Mann-Whitney test, Bonferroni correction for 6 comparisons) are indicated with blue asterisks and a light blue background tint. (A) RNAi of *ogre* (*Inx1*). The GF responds significantly more strongly to SEP1 with amplitudes 0.3–0.4 mV. (B) RNAi of *Inx2*. There is no significant change in the response of the GF. (C) For RNAi of *Inx3* there is no significant change in the response of the GF. (D) RNAi of *zpg* (*Inx4*). There is no significant change in the response of the GF. (E) For RNAi of *Inx5* there is no significant change in the response of the GF. (F) RNAi of *Inx6*. The GF responds significantly more strongly to SEP1 of 0–0.3 mV amplitudes and to SEP2 of 0–0.4 mV amplitudes. (G) RNAi of *Inx7*. There is no significant change in the response of the GF. (H) RNAi of *shakB* (*Inx8*), using the Bloomington HMC04895 line. Only for this innexin is there a significant decrease in the response of the GF, to SEP1 of greater than 0.2 mV and SEP2 greater than 0.4 mV. For full genotypes see S1 Table.

**Fig 5:**
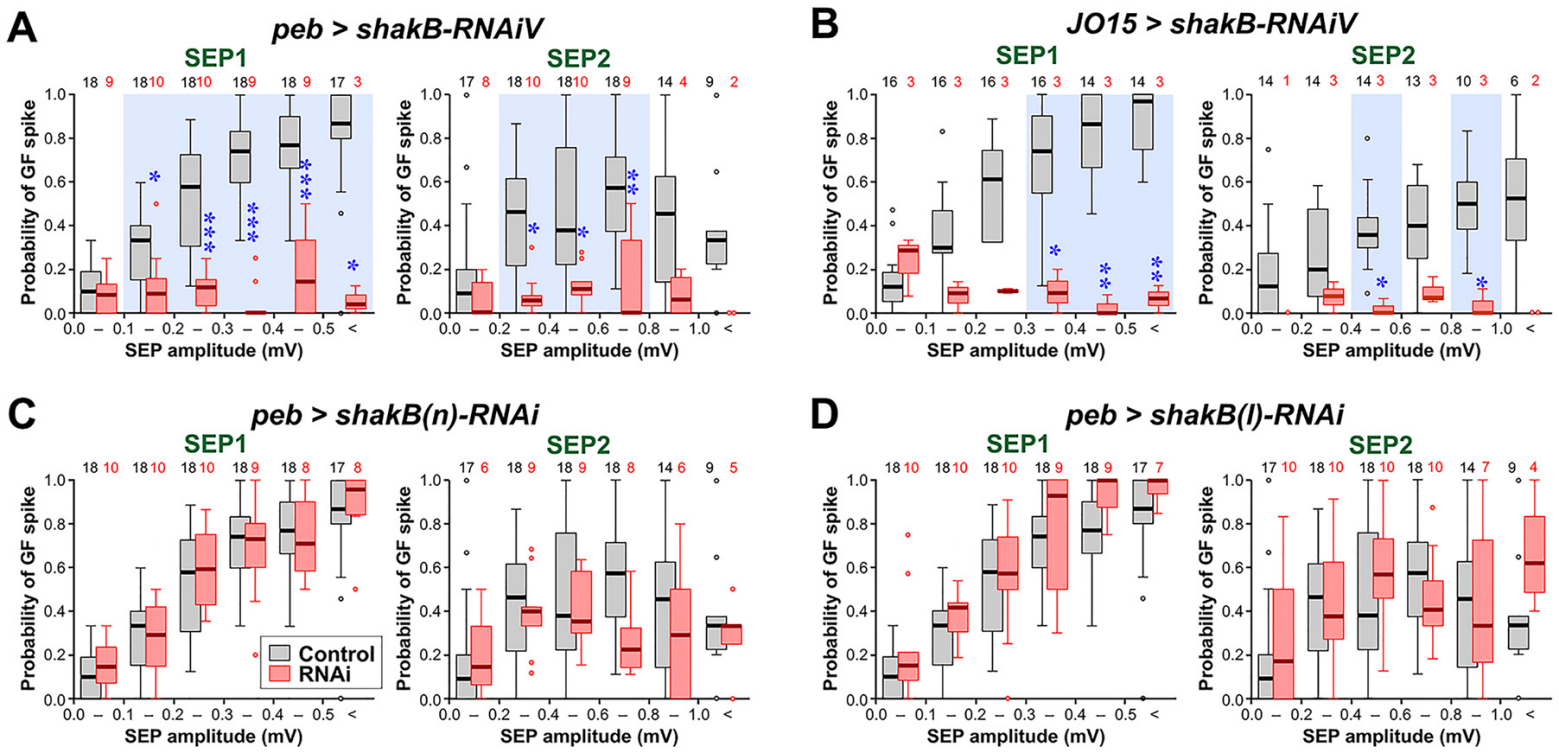
ShakB knockdown with different RNAi lines and drivers. Box-whisker plots of the probability of GF spiking in response to SEP1 and SEP2. Controls are shown in gray, experimental data in pink. Statistically significant differences between RNAi versus control (Mann-Whitney test, Bonferroni p-correction for 6 comparisons) are indicated with blue asterisks and a light blue background tint. (A) The Vienna GD12666 line (*shakB-RNAiV*) strongly decreases the GF response to both SEPs. (B) The same line, driven by *JO15-GAL4* (without Dcr-2) in auditory JONs alone, is also effective at inhibiting the GF response. (C) The JF02603 line, which targets *shakB(n)* (*shakB(n)-RNAi*) has no effect on the JON-GF connection. (D) The JF02604 line, which targets *shakB(l)* (*shakB(l)-RNAi*) also has no effect on the JON-GF connection. For full genotypes see S1 Table.

**Fig 6:**
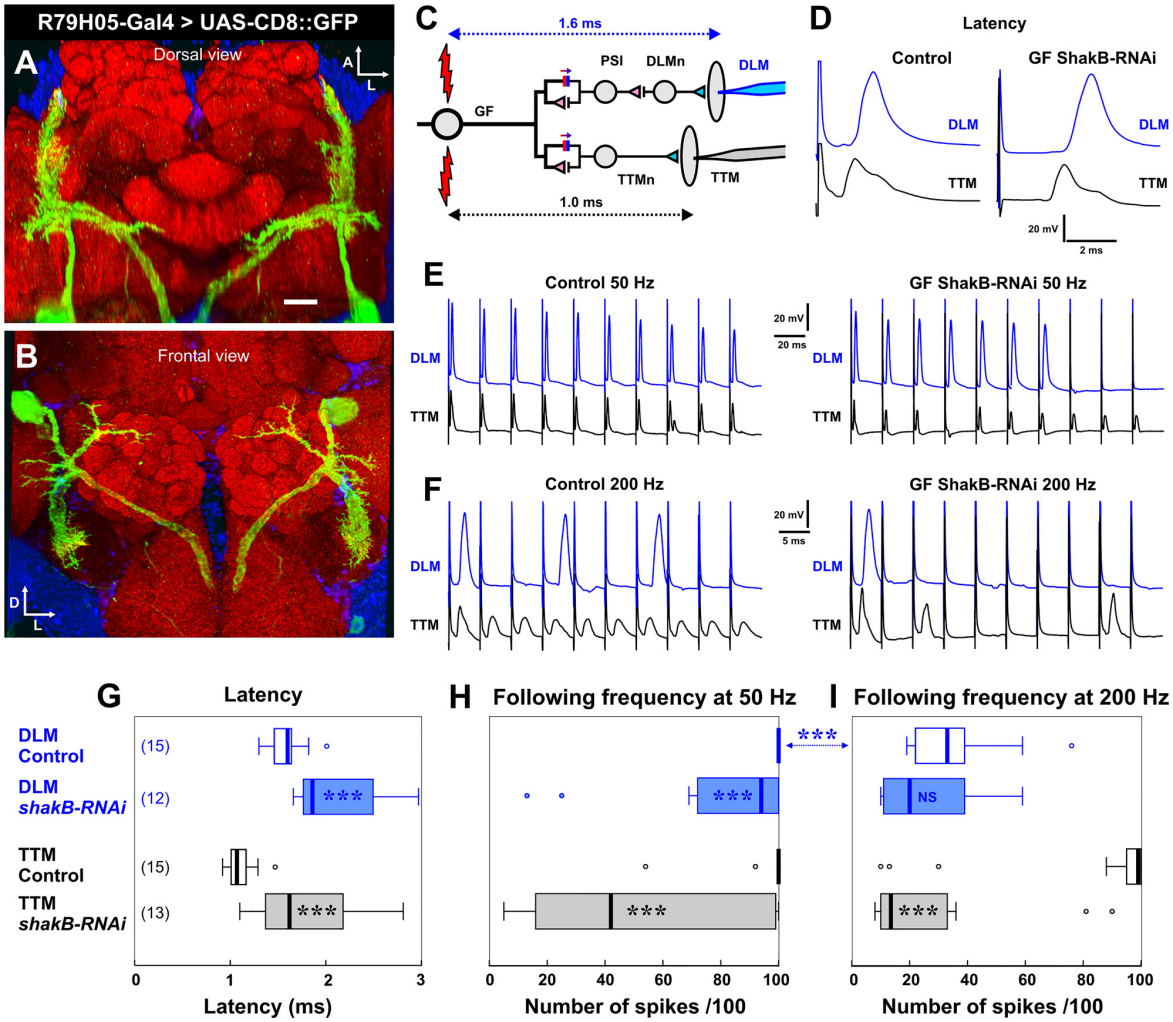
ShakB RNAi driven in the GF alters transmission at its output synapses. (A) Dorsal and (B) frontal views of a complete confocal stack of the GF in the brain, stained by CD8::GFP driven by the *R79H05-GAL4* driver. Scale bar is 20 μm. (C) Circuit diagram of the outputs of the GF, indicating the recording electrodes in the DLM (blue) and TTM (black) muscles. The GF is stimulated directly by high voltage across the eyes. The approximate latency between this stimulus and the muscles responses is indicated. (D) Example traces illustrating the increased latency in an animal where *shakB-RNAi* is driven by *R79H05-GAL4*. (E) Examples of traces showing how *shakB-RNAi* in the GF decreases the ability of DLM to follow the stimuli at 50Hz. (F) Examples of traces showing how *shakB-RNAi* in the GF decreases the ability of both DLM and TTM to follow the stimuli at 200Hz. (G-I) Box-whisker plots showing significant effects of *shakB-RNAi* on the latency (G) and following frequency (H, I) of the muscle responses (Mann-Whitney test). For full genotypes see S1 Table.

N represents the number of animals. The normality of the distribution of the data sets was first determined then subsequent tests were carried out using PAST3 software [33]. To identify significant differences between means of control vs. experimental groups, normally-distributed data were compared with t-tests, while non-normally distributed data were compared using a Mann-Whitney U test. In both cases a Bonferroni correction for multiple comparisons was applied. In figures, * denotes p ≤ 0.05, ** p ≤ 0.01, *** p ≤ 0.001. Box-whisker plots were prepared using BoxPlotR (boxplot.tyerslab.com), using the Tukey whisker definition. For each plot in Figs 2–6, center lines show the medians; box limits indicate the 25th and 75th percentiles as determined by R software; whiskers extend 1.5 times the interquartile range from the 25th and 75th percentiles; outliers are represented by points.

## Results and Discussion

In order to reduce selectively innexin expression in presynaptic JONs only, we used a RNA interference (RNAi) strategy [18] under Gal4-UAS control. As in our previous study [14], we used a Gal4 driver that is expressed strongly in JONs, namely *pebbled* (*peb*) *-GAL4*, which shows very strong expression in all sensory neurons but not in central neurons [20], and begins to be expressed soon after post-mitotic JONs have entered the CNS [14]. For all of the long hairpin (VALIUM10) experiments, Dicer-2 (Dcr-2) was added to facilitate the production of short interfering RNA fragments and thereby increase the strength of the knockdown [34]. However, the *ogre-RNAi*, *Inx2-RNAi*, *Inx6-RNAi and shakB-RNAi (HMC04895)* constructs already produce short hairpin RNAs [35] and so Dcr-2 was not required. Some of the Inx2 and Inx6 RNAi experiments had Dcr-2 included anyway but, since it had no significant effect, data were combined with those from experiments without Dcr-2. When *ogre-RNAi* was driven with *peb-GAL4* it was lethal, so *JO15-GAL4* [36] was used for the *ogre-RNAi* experiments. This strongly-expressing driver is restricted to A and B-type JONs, those which have been primarily associated with sound detection [37,38], and scattered central neurons. We were not able to use it for all the experiments because, as we observed previously, when combined with *UAS-Dcr-2* it reduces the amplitude of sound-evoked potentials [14]. Fortunately in the particular case of *ogre*, the RNAi construct did not require Dcr-2.

### Effects of Innexin knockdown on JON response to sound

As an essential part of the electrophysiological experiments to assay the strength of the JON-GF connection, we measured the amplitude of sound-evoked potentials (SEPs) generated by JONs in the antennal nerve. These SEPs are extracellularly recorded compound action potentials, so their amplitude is presumably proportional to the number of JONs that are firing an action potential in synchrony. It should be noted that the amplitude of SEP1 is always about 0.66x smaller than that of SEP2 at any given sound intensity (Fig 2A)–this is simply due to the fact that the sound stimulus waveform was designed to have a gradual onset in order to prevent a JO response to transient air acceleration. We considered the two SEPs separately since we have no evidence that identical populations of JONs contribute action potentials to each one, and because the response of the GF to each is different (see below).

Notwithstanding the smaller numbers of experimental animals in some cases (Ogre and Inx7) there were significant effects on SEP amplitude with RNAi of five of the innexins: Ogre, Inx2, Inx3, Inx6 and ShakB. The first four showed a reduction in amplitude, either for high intensity sounds (Ogre) or for low to medium intensity sounds (Inx2, Inx3, Inx6). Conversely, ShakB RNAi resulted in a 30% increase in SEP2 amplitude for higher intensity sounds. There remains the possibility that some of these differences may still be false positives, so these results should be regarded as exploratory and as the basis for a more detailed investigation in the future. Regarding the specificity of the RNAi effects, it should be noted that the short hairpin constructs used for knockdown of Ogre, Inx2, Inx6 and ShakB have no or few putative off-targets (OTE program at FlyRNAiorg versus dscheck.rnai.jp [39,40]: see S2 Table). However, the Inx3 RNAi construct may have potential off-target effects which could conceivably account for its effects on SEP amplitude. Despite these caveats, these results raise the possibility that these innexins may play a role in the excitability or sound-responsiveness of subsets of JONs. Another possibility is that, since the driver is first expressed soon after the JONs have entered the CNS, the development of the JONs is adversely affected, although we saw no anatomical changes in the distribution or branching patterns of the JON axons (see S2 Fig). In this study it was not technically feasible to carry out quantitative RT-PCR to measure the extent of the RNA knockdowns. The only construct for which we observe no effect of any kind (including conditional pupal lethality) is that for Inx7 RNAi, for which we cannot discount the possibility that it is ineffective.

Because of the results on the JON-GF synapse (see below), we focused in more detail on ShakB. The *shakB*^*2*^ mutation, which removes the ShakB(N) and (N+16) isoforms but not ShakB(L), had no effect on SEP amplitude (S1B Fig). Other *shakB-RNAi* lines did have significant effects: the Vienna line GD12666 which, like the Bloomington HMC04895 used in Fig 2I, should target all transcripts, substantially reduced SEP amplitudes (S1C Fig). However, it must be noted that, whereas all the other RNAi lines used in the study are inserted at attP2 on chromosome 3, the P-element insertion site of the Vienna construct is not known—this raises the possibility that it could therefore have additional effects on the neurons, as well as potential off-target effects that HMC04895 does not have (S2 Table). Two other Bloomington constructs (JF02603 and JF02604) were also used to target different isoforms of *shakB* RNA. In *Drosophila*, RNAi is isoform specific [41] so, according to Flybase, JF02603 should only affect the RC and RD transcripts, with RC corresponding to ShakB(N) (S1D Fig). JF02604 should only affect the RA and RE transcripts, with RA corresponding to the ShakB(L) isoform (S1E Fig). Expression of either of these constructs in the JONs also resulted in significant reductions in SEP amplitude. However, the *shakB*^*2*^ mutation has no effect, which means that the effect of JF02603 cannot be due to *shakB(n)* RNA knockdown, but must either be due to knockdown of the hitherto uncharacterized *shakB-RD* transcript or of an off-target (see S2 Table for list).

With the caveat that we do not know the extent of RNA knockdown, the exploratory RNAi results described above suggest that Ogre, Inx2, Inx3 and Inx6 (and possibly ShakB(L)) might be involved somehow in the response of the JONs to sound. It will be necessary to eliminate the possibility that they are simply required for JON development by using drivers with temporally restricted expression (or by using temperature-sensitive Gal80 to restrict it [42]), using the newer generation, more specific, VALIUM20 constructs as they become available. Immunostaining of the antennal nerve does indicate that Inx2, and perhaps Ogre, may be present (see S3 Fig), supporting the possibility that these particular innexins have a function in adult JONs. Since the SEP is a compound action potential, its amplitude will depend on the synchronization of spikes in several JONs, a process that would certainly be facilitated by gap junctional coupling between the axons. This effect would be particularly important for SEPs of smaller amplitudes, where fewer axons are firing in synchrony. Indeed, ultrastructural gap junctions have been observed between JON axons, which first prompted the suggestion that they are required for spike synchronization [43]. Chemical synapses cannot be involved in this process, since it has been shown that blocking synaptic transmission leaves the SEPs unaffected [44].

### ShakB is required in JONs for the GF to respond to sound

As established in our recent study [14], we used an indirect method to record the probability of the GF firing an action potential in response to SEPs of different amplitudes. The recording configuration is illustrated in Fig 3A. Sound-evoked synaptic input alone is not normally sufficient to bring the GF to spike threshold, so to enable a response the SEPs must be paired with an eye stimulus. A low voltage stimulus across the eyes (Fig 3B1) evokes the long-latency response of the GF, which has long been presumed to be due to direct stimulation of a series of visual interneurons that eventually input onto the GF lateral dendritic field [10,30,45–47]. However, this idea has never been rigorously tested with modern genetic tools for neuronal silencing/activation. This suprathreshold eye stimulus is then reduced slightly so as to be just subthreshold (so that one or fewer action potentials are elicited per 10 trials: Fig 3B2), then its timing is adjusted so that it summates with the input from either SEP1 or SEP2 (Figs 3B3 and 4). The GF response to each in turn can then be measured, at different sound intensities.

As in the previous study, we find that, in controls, the smaller SEP1 paradoxically elicits a stronger response in GF than does SEP2, particularly at large amplitudes (Fig 3C). The reason for this ‘depression-like’ effect is not yet fully understood, however previous tetanus toxin experiments showed that it is not due to the chemical component of the synapse, and that it is greatly reduced (ie. the GF response is increased) by the formation of more synaptic contacts brought about by ectopic expression of the transcription factor Engrailed in JON afferents that do not normally contact the GF [14].

The only innexin for which RNAi knockdown greatly reduced the strength of the JON-GF connection was ShakB (Fig 4H). The median response probability was reduced to baseline levels (our experimental paradigm means that a firing probability of 1 in 10 action potentials could simply be due to spontaneous background activity). These results provide strong evidence that electrical synapses between the JONs and the GF require ShakB in the presynaptic neuron, and are thus presumably composed of ShakB on both sides.

Surprisingly, knockdown of two of the other innexins, particularly Inx6 (Fig 4F) and to a lesser extent Ogre (Fig 4A) had the somewhat paradoxical effect of strengthening the JON-GF connection. Both of these also decrease SEP amplitude (Fig 2), so one way in which this could happen is if these particular innexins are differentially expressed in a sub-population of JONs that do not actually connect to the GF, i.e., are not expressed in the A-type JONs. In this case, knockdown would de-synchronize this subpopulation and decrease the overall amplitude of the SEPs (which are recorded from the population as a whole) but leave unaffected the JON-GF connection. The strength of the JON-GF connection itself would remain the same, but the GF response would be elicited by an apparently smaller input, thus accounting for our results.

### RNAi of *shakB(n)* and *shakB(l)* has no effect on the JON-GF synapse

The inhibitory effect of ShakB knockdown was also apparent for the other RNAi line from Vienna, which targets all isoforms (Fig 5A). As a control for the driver, ShakB knockdown was also effective when driven by *JO15-GAL4* (alone, without Dcr-2: Fig 5B) which is restricted in its expression to only the auditory neurons of group A [48], those that normally connect to the GF, and also the group B neurons, which do not. In contrast, the two other Bloomington RNAi lines, JF02603, which targets the *shakB(n)* and *shakB-RD* transcripts, and JF02604, which targets *shakB(l)* and *shakB-RE*, had no effect on the JON-GF response (Fig 5C and 5D). These constructs appear to be functional to some extent, since both of them reduce SEP amplitude (see previous section), although we cannot rule out a chance of additional off-targets being affected (see S2 Table). The ineffectiveness of these lines in inhibiting JON-GF transmission suggests that neither ShakB(N) or ShakB(L) is required presynaptically. We can deduce from these results that it is the remaining known isoform, ShakB(N+16), that is involved at the synapse. Direct confirmation of this however would require (N+16)-specific knockdown, which is unfortunately not feasible with the currently available RNAi tools.

The GF is thought to express ShakB(N+16) at its output synapses in the thorax, and at its synapses with the GCIs (see Fig 1) [5]. It would be expected that it also expresses this isoform at its synapses with the JONs, although this assumption is perhaps unjustified in view of the fact that olfactory interneurons can form ShakB gap junctions at some of their output synapses but not others [49]. However, *shakB(n+16)* is indeed the only transcript from *shakB* detectable in the GF [5]. Thus, in this case, the JON-GF synapses would be homotypic (the same isoform on both sides) and, therefore, the prediction from heterologous expression studies is that they would be non-rectifying [5]. The GF is known to be capable of producing both calcium- and sodium-dependent action potentials (in the soma and axon, respectively) [6,50], and it is quite strongly electrically coupled to the JON axons [13]. If the JON-GF synapses are indeed non-rectifying, we would predict that GF spikes could perhaps be transmitted retrogradely to the JON somata. Of course, one of our original questions that provoked this study remains open: why do the GF-GCI synapses, which are also thought to be homotypic ShakB(N+16) [5], pass both LY and NB, while the JON-GF synapses (which from the data in the present study appear to have the same composition) do not pass LY?

### RNAi knockdown of ShakB in the postsynaptic GF affects its outputs in the thorax

A good positive control for ShakB RNAi would be to test its effects when expressed in the postsynaptic cell, the GF. We could not test the effect of ShakB RNAi in the GF at the JON-GF synapses themselves, because our assay depends on the integrity of the thoracic outputs of the GF, which would also be disrupted by loss of ShakB. Instead, we used the conventional assays of function that measure latency and following frequency of the GF thoracic outputs to the TTM and DLM motorneurons [31]. In a screen of Janelia drivers we identified GMR79H05 as strongly, and cleanly, expressing in the GF. Thus we did not utilize the commonly used *A307* and *c17* Gal4 drivers, which are either less specific for the GF, or relatively weak, respectively. GMR79H05 contains a fragment of an enhancer close to the *thisbe* gene. When used to drive CD8::GFP it can be seen to strongly label the GF in the brain (Fig 6A and 6B), with no labeling of the TTMn in the thorax. We then stimulated the GF directly (by increasing the voltage across the eyes) so as to achieve a “short-latency” response in the TTM and DLM (Fig 6C and 6D). In control animals (genotype *UAS-Dcr-2/+; R79H05-GAL4/+)*, at our laboratory temperatures, this latency is approximately 1.0 and 1.6 ms, respectively. ShakB knockdown results in significant increases in latency of both TTM and DLM responses (Fig 6G). The second test of synapse function is that of following frequency, where the GF is repeatedly stimulated at different frequencies and the number of responses is counted. The GF-TTMn connection in controls can follow one-to-one at 200 Hz or less (Fig 6F), whereas the GF-PSI-DLMn pathway is able to follow at 50Hz (Fig 6E), but not at 200Hz (Fig 6F). RNAi of ShakB in the GF significantly reduced the following frequency of TTM at both 50 and 200 Hz, and of DLM at 50 Hz (Fig 6E–6I). These results are comparable to those obtained with the *shakB*^*2*^ null mutants [5], and are consistent with ShakB RNAi abolishing the electrical component of synaptic transmission at the mixed electrical/chemical synapses between the GF and TTMn and PSI, and provide independent confirmation of its functional efficacy.

### Neurobiotin-coupling between JONs and the GF is prevented by ShakB RNAi in JONs

**Fig 7:**
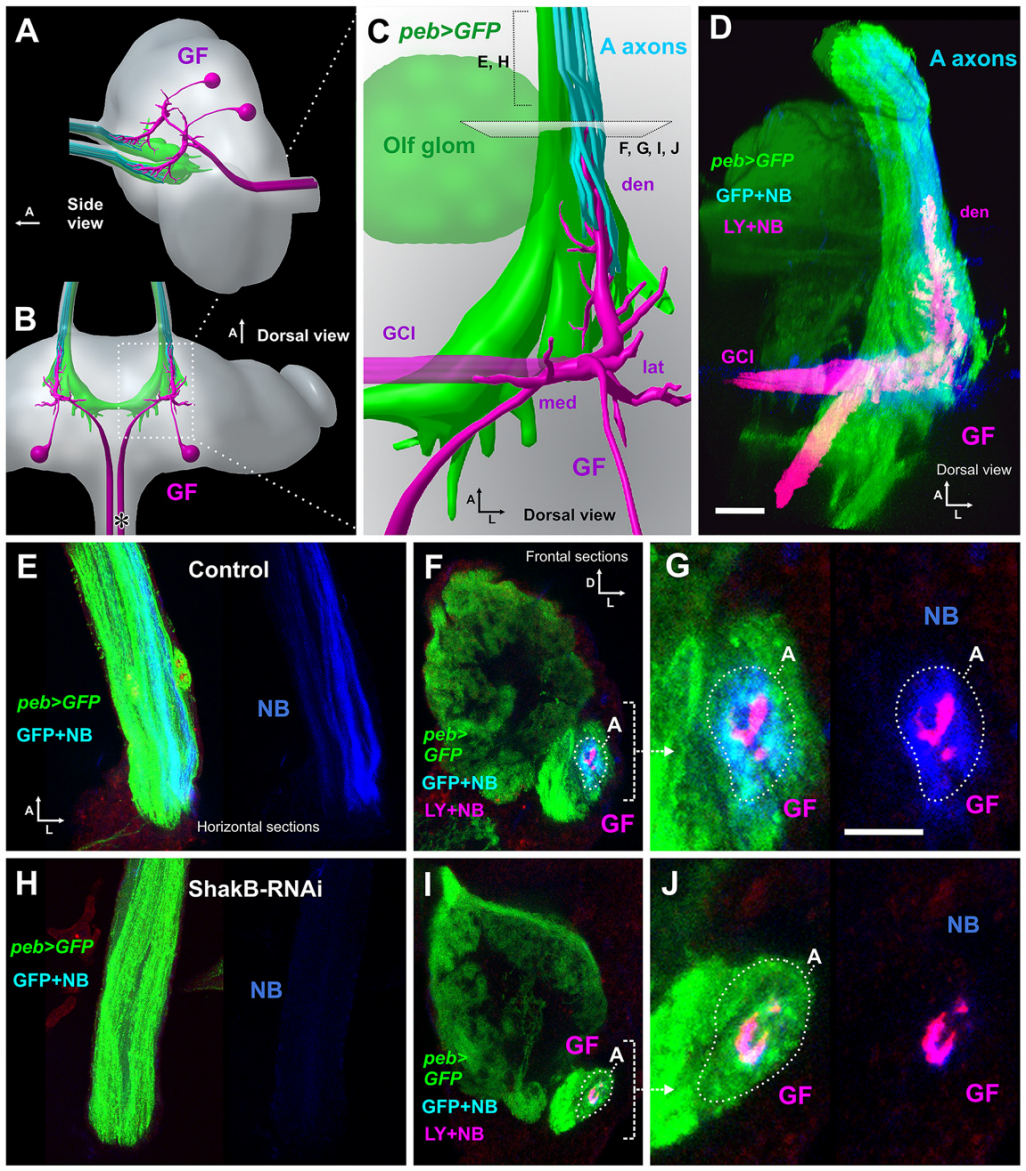
ShakB RNAi prevents NB coupling of JONs with GF. In these animals, *peb-GAL4* was used to drive expression of CD8::GFP, and also Dcr-2. (A, B) Diagrammatic views of the brain, showing the pair of GF neurons (magenta) in relation to the antennal afferent projection (green and cyan). (A) Oblique side and (B) dorsal views, arrows indicate anterior (A). The asterisk in B indicates the approximate site of GF axon impalement for dye injection. (C) Enlarged dorsal view of the GF dendrites, showing the anterior large dendrite (den) inserted within a cylinder of JO-A axons, to which they are NB-coupled (and hence appear cyan), and also the lateral (lat) and medial (med) dendrites. The olfactory glomeruli, also labeled by *peb-GAL4*, are indicated (olf). (D) Dorsal view of a complete confocal stack from a control animal, in which *peb-GAL4* drives expression of CD8::GFP (green) and Dcr-2 in JON axons (and other sensory neurons—for clarity, olfactory axons are shown as partially transparent). Neurobiotin (NB: blue) and Lucifer Yellow (LY: red) are injected into the GF (making it appear magenta). NB alone transfers across gap junctions into the green JO-A axons (to appear cyan). Both LY and NB transfer into the commissural interneurons (GCI). (E) Projection from 3–5 horizontal sections through the antennal nerve of a control animal, showing peb-driven GFP and a subset of axons containing NB. (F) Projection of 3 confocal sections taken from the anterior end of the GF dendrite shown in D, showing its insertion within a ring of several GFP-expressing JO-A afferents (A), to which it is NB coupled. (G) Higher magnification view of the NB-coupled A afferents. In the right panel, the green channel is omitted for clarity. (H-J) Animal in which *peb-GAL4* drives CD8::GFP, Dcr2 and *shakB-RNAi*. (H). Section through the nerve, showing absence of NB coupling in the axons. (I) Low- and (J) high-power views of the A group of JON axons surrounding the GF dendrite, showing the absence of NB coupling. For full genotypes see S1 Table. Scale bar in D: 20 μm in D, E, F, H, I; Scale bar in G: 10 μm in G and J.

The electrophysiological results suggest that presynaptic ShakB is required for the JON-GF synaptic connection to function. If this is the case, it should also be required for the Neurobiotin (NB) dye coupling that we described previously [14]. We injected NB and LY into the GF axon in animals in which the JON axons (and other sensory axons) were labeled with CD8::GFP driven by the *peb-GAL4* driver (along with Dcr-2). As before, we found that, in control animals, NB passes retrogradely into a subset of JON axons (belonging to the “A” group [48]), whereas LY does not (Fig 7D–7G). RNAi knockdown of ShakB (all isoforms) completely abolishes this NB coupling (Fig 7H–7J), consistent with it disrupting the formation of gap junctions with the GF. In 4 additional experiments, we also observed that *R79H05-GAL4*-driven *shakB* RNAi (ie. in the GF alone) also prevents NB coupling (S2A Fig). Finally, driving JF02603 (ShakB(N)), or JF02604 (ShakB(L)) in the sensory axons does not affect dye coupling with the GF (S2B and S2C Fig), suggesting that it is the (N+16) isoform that is required.

### Plaques of ShakB immunostaining at the JON-GF contacts are abolished by RNAi

**Fig 8:**
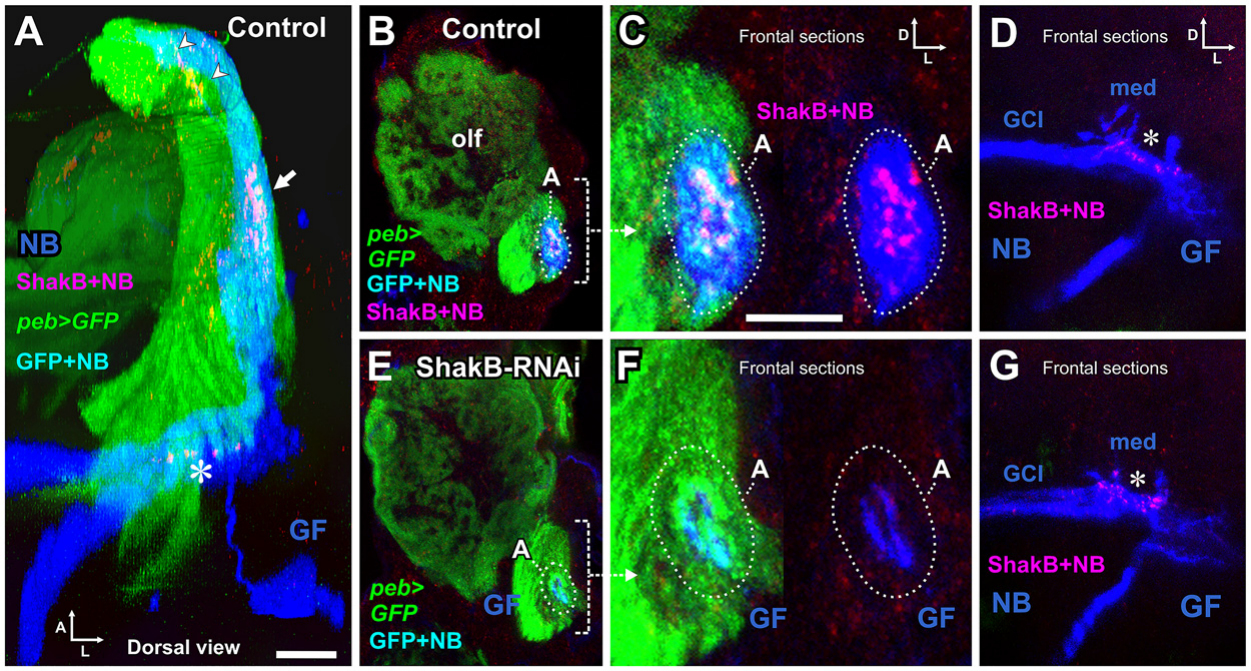
ShakB immunostaining and RNAi knockdown. (A) Dorsal view of a complete confocal stack of a control animal, in which *peb-GAL4* drives expression of CD8::GFP (green) and Dcr-2 in JON axons (and other sensory neurons—for clarity, olfactory axons are shown as partially transparent). Neurobiotin (NB: blue) is injected into the GF and transfers across gap junctions into the green JO-A axons (to appear cyan). The red channel shows immunostaining for the gap junctional protein ShakB, which forms plaques in the JON-A axons (arrow) and on the medial GF dendrite (asterisk). Scattered staining is also present in the antennal nerve (arrowheads). (B) Maximum intensity projection of 3 frontal slices, showing *peb*-driven GFP staining in olfactory glomeruli (olf) and JON axons. The A group of the latter is indicated (dotted oval), showing NB dye transfer (cyan). (C) High power view of the A axons, showing the ShakB plaques overlying the NB staining (magenta), which are seen more clearly in the right panel where the green channel is omitted. (D) Projection of 20 frontal sections through the GF medial dendrites (med) and coupled giant commissural neurons (GCI). ShakB plaques (magenta) are present on the medial GF dendrite (asterisk). (E-G) Experimental animal in which *peb-GAL4* drives expression of CD8::GFP, Dcr-2 and *shakB RNAi*. (E, F) ShakB staining is not present around the NB-filled GF dendrites, nor does NB pass into the A axons (dotted oval). (G) ShakB staining on the GF medial dendrite is unaffected by RNAi in the JONs. For full genotypes see S1 Table. Scale bar in A: 20 μm in A, B, D, E, G; Scale bar in C: 10 μm in C and F.

For immunostaining of ShakB protein (all isoforms), we injected NB into the GF axon in animals with *peb-GAL4* driving GFP and Dcr-2 as above, but with the omission of LY in the GF. These animals were then processed for ShakB immunostaining (Fig 8). In control animals, clusters of large plaques or puncta of ShakB immunoreactivity were located at the end of the GF dendrite, where it makes contact with JON axons to which it is NB-coupled (Fig 8A–8C). These ShakB puncta are approximately 1 μm in diameter, and are thus similar in size to those described at the GF-TTMn synapses [8]. Further towards the posterior, ShakB puncta are also visible along the medial dendrite of GF, where it contacts the giant commissural interneurons (GCI), to which it is also dye-coupled (Fig 8D). In addition, there was faint scattered punctate staining throughout the antennal nerve (Fig 8A and S3A Fig). In presynaptic *shakB-RNAi* animals (both the Bloomington and Vienna lines), the ShakB staining between the JONs and GF was abolished, along with the NB dye coupling (Fig 8E and 8F, S3B and S3C Fig). As a useful internal control, in these same RNAi animals the ShakB staining at the medial GF dendrite was unaffected (Fig 8G). Thus, presynaptic expression of *shakB* in the JONs is necessary for the formation of ShakB-containing junctions with the GF dendrite, and for the dye coupling that they permit. In three animals, *peb-GAL4* was used to drive *shakB(n)-RNAi* (JF02603) and *shakB(l)-RNAi* (JF02604); ShakB antibody staining was unaffected (S2D and S2E Fig).

We obtained antibodies against Ogre, Inx2, and Inx7, and carried out immunostaining of the antennal nerve and region of contact of the JONs with GF. No other innexin formed patches of staining around the GF dendrite as did ShakB, however, patches of Ogre and Inx2 immunoreactivity appeared to be present in the antennal nerve (see S3D and S3G Fig). This, combined with the effect of knockdown of these innexins on SEP amplitude, suggests that they might be involved in coupling between JON axons. No Inx7 staining was observed, which correlates with its knockdown not having any effect on the SEP electrophysiological assays.

### Presynaptic overexpression of ShakB isoforms alters the formation of JON-GF synapses

**Fig 9:**
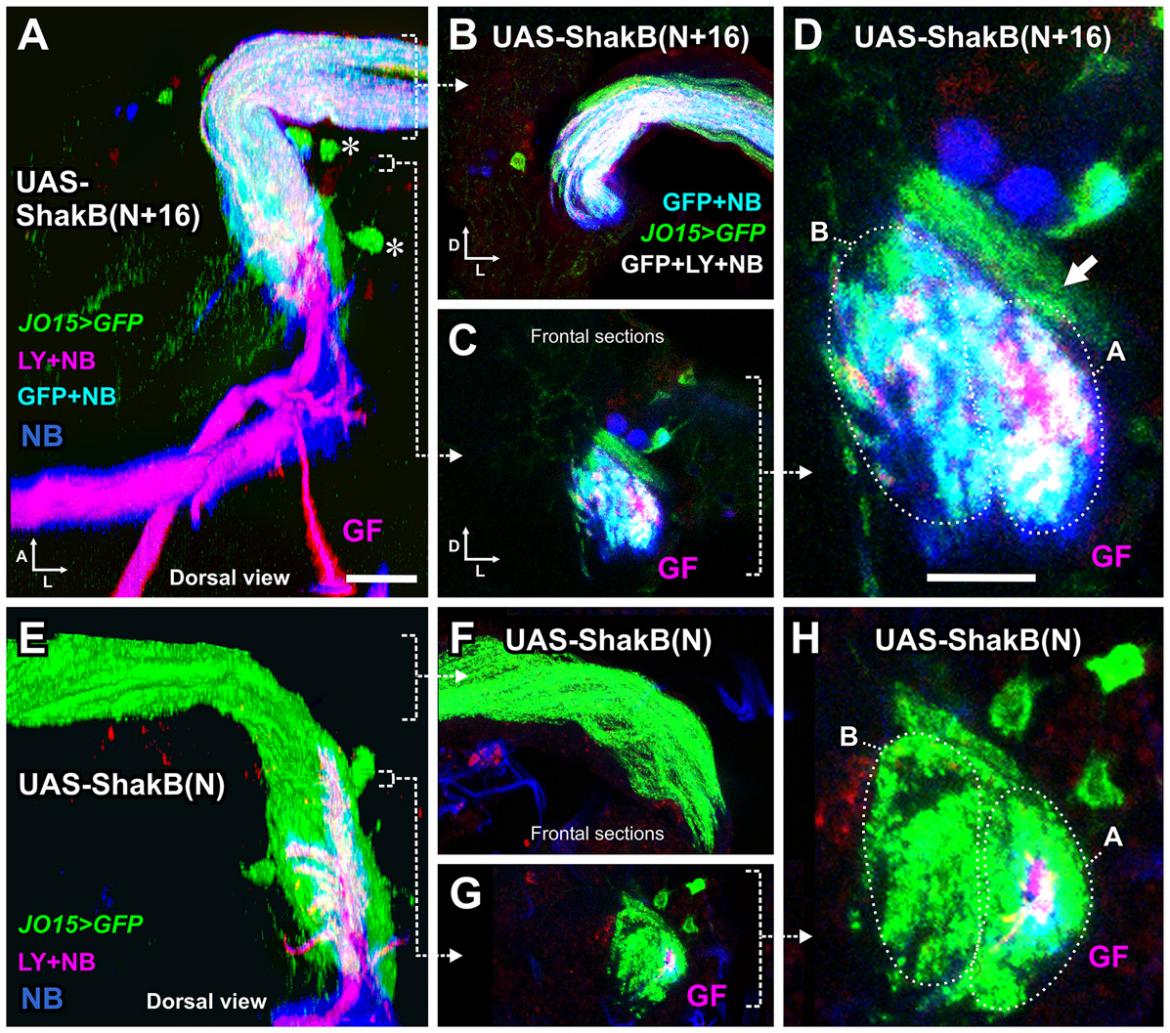
Expression of different isoforms of ShakB in JON axons alters dye coupling to the GF. (A-D), *JO15-GAL4* drives expression of *UAS*-*ShakB(N+16)* in JO-A and JO-B neurons, along with CD8::GFP to label the axons. LY and NB are injected into the GF and NB is transferred across gap junctions into the JON axons. Some LY also appears to be transferred. (A) is a dorsal view of a 3D reconstruction, (B-D) are Z projections of frontal slices. (B) is a projection of several slices through the nerve, in order to show the NB and LY within the axons. (C) and (D) are low and high power views of slices through the axons in the region of the GF dendrite. (D) The A and B groups of axons are indicated (dotted ovals), along with another group of peripheral A axons that do not dye couple (arrow). (E-H) *JO15-GAL4* drives expression of the ShakB(N) isoform and abolishes gap junctional coupling. (F) Projection of sections through the nerve, showing the absence of coupling (compare with B). (H). High power frontal section through the axons in the region of the GF dendrite, showing only the GF filled with both NB and LY. For full genotypes see S1 Table. Scale bar in A: 20 μm in A, B, C, E, F, G; Scale bar in D: 10 μm in D and H.

The RNAi experiments indicate that it is the ShakB(N+16) isoform, and not ShakB(N) or ShakB(L), which is required presynaptically for functioning of the JON-GF synapse. As another way of testing this, we overexpressed ShakB(N+16) and ShakB(N) [5] in auditory neurons using the *JO15-GAL4* driver. In these animals, CD8::GFP was also expressed in the auditory axons, and Lucifer Yellow (LY) was injected into the GF to delineate its morphology, along with NB to assay dye coupling. Normally, the GF is NB-coupled only to the A group of JON axons (see Fig 7) [14]. With overexpression of ShakB(N+16), we found large amounts of NB coupling in both A and B groups of axons (Fig 9A–9D), along with some LY coupling which is not normally observed.

This result is reminiscent of the effects of Engrailed overexpression using the same driver, where ‘inappropriate’ synaptic connections with B axons are formed [14], and implies that ShakB(N+16) expression might have an instructive role in the recognition of synaptic partners. The idea that gap junctions are involved in determining synaptic specificity is a fairly old one [16], but experimental evidence has been lacking until more recently. Both Ogre and ShakB(N) are required during pupal development in retina and lamina neurons, respectively, for the proper formation of the histaminergic synapses that connect them in the adult [12]. Ectopic expression of leech innexin 6 in neurons that do not normally express it is also sufficient to alter their pattern of connections to other neuronal networks [51,52], and innexin 1 is required for contact-dependent homolog avoidance [53]. Finally, insertion of ectopic connexins rewires *C*. *elegans* chemosensory circuits and alters behavior [54].

It is still not clear why, if ShakB(N+16) is the endogenous presynaptic isoform, its overexpression should allow LY coupling into the axons. One possibility is that the selectivity for NB versus LY of ShakB(N+16) homotypic channels is not absolute. Thus with large amounts of LY present, and with the formation of a stronger gap junctional connection than normal, some LY dye transfer can take place. Another, more speculative possibility is that the dye selectivity of the gap junctions depends not just upon their innexin composition but on some additional presynaptic component or process, such as phosphorylation [1]. This could also explain why GF-GCI synapses pass LY but JON-GF synapses normally do not. In the case of presynaptic ShakB(N+16) overexpression such a mechanism could become saturated, leaving some of the excess junctions unmodified and thus less selective against LY.

Conversely, and somewhat surprisingly, expression of ShakB(N), ie. the ‘wrong’ isoform, in A and B JONs, led to the almost complete abolition of dye coupling between JON-A axons and the GF (Fig 9E–9H). This dominant negative effect of ShakB(N) overexpression gives some support to the idea that innexin expression could actually have some instructive role in synaptic partner choice. However, the sequences of ShakB(N) and (N+16) are identical except for the eponymous 16 amino-acids on the N-terminus of the latter, which is intracellular [15], making it impossible for this portion of the protein to participate directly in cell-cell adhesion. The N-terminus of ShakB is however known to be important for voltage sensitivity, and also probably for trafficking and oligomerization [55]. There remains the possibility of it being required for an indirect role in target recognition, such as recruitment to a multiprotein complex containing adhesion molecules. However, for Inx2 and 3 in epithelia, where this phenomenon has been demonstrated, it is the C-terminus that is important [56,57]. At GF output synapses ShakB gap junction formation is closely coupled to Netrin-Frazzled signaling [58], so one possibility remains that the N-terminus is important for a similar process in the formation of JON-GF connections.

The only prior example of a similar dominant negative effect of a gap junctional protein in the literature appears to be that of a vertebrate connexin, Cx33, which suppresses the activity of other connexins in the testis [59] by promoting their sequestration to early endosomes [60]. We note that ShakB(N) is not able to formed homotypic gap junctions in paired oocytes [2], and has not yet been found to interact with any other innexin to form functional heterotypic or heteromeric junctions. It seems possible that ectopically expressed ShakB(N) sequesters, or otherwise inactivates, the resident ShakB(N+16) in JONs to inactivate gap-junction communication with GF. In the future, we could perhaps begin to investigate the putative instructive function of ShakB by determining whether ectopic isoform expression also affects the distribution of the chemical component of the JON-GF synapse, as does Engrailed misexpression [14].

## Supporting Information

S1 Fig Effects of shakB mutation and knockdown on the amplitudes of sound-evoked potentials. (A) Sample traces of sound-evoked potentials (SEPs) recorded from the base of the antenna, in response to 200 Hz sound pulses. (B) There is no significant difference in amplitudes of SEP1 and SEP2 in *shakB*^*2*^ mutants compared to controls (*peb-GAL4 > Dcr-2*). (C-E) Amplitudes of SEP1 and SEP2 in animals with different RNA interference constructs targeting *shakB*, compared to controls in which the RNAi was omitted. Paired t-tests or Mann-Whitney tests (with a Bonferroni correction for 3 comparisons) were used to determine significant differences from control. These differences are indicated with blue asterisks and a light blue background tint. (C) *peb-GAL4* driving *shakB-RNAi GD12666* (Vienna line, long hairpin) and *UAS-Dcr-2*. Both SEPs are reduced by about half. (D) *peb-GAL4* driving *shakB-RNAi JF02603* (targeted against *shakB(n)*, long hairpin) and *UAS-Dcr-2*. Both SEPs were reduced in amplitude. (E) *peb-GAL4* driving *shakB-RNAi JF02604* (targeted against *shakB(l)*, long hairpin) and *UAS-Dcr-2*. Both SEPs were reduced in amplitude. For full genotypes see S1 Table.
(TIF)

S2 Fig ShakB RNAi in GF prevents dye coupling; presynaptic N and L isoform RNAi leaves dye coupling and ShakB immunostaining unaffected. Each figure is a single frontal confocal slice. (A) ShakB-RNAi was driven by the *R79H05-GAL4* driver in the GF. Dye coupling into A axons is prevented. (B-E) In these animals, *peb-GAL4* was used to drive expression of CD8::GFP, and also Dcr-2. (B) ShakB(N) RNAi with JF02603. LY and NB were injected into the GF. NB coupling into A-type JONs is unaffected. (C) ShakB(L) RNAi with JF02604. LY and NB were injected into the GF. NB coupling into A-type JONs is unaffected. (D) ShakB(N) RNAi with JF02603. NB was injected into the GF and the preparation was stained with ShakB antibody. NB coupling into A-type JONs is unaffected, as were the ShakB plaques surrounding the GF dendrite. (E) ShakB(L) RNAi with JF02604. NB was injected into the GF and the preparation was stained with ShakB antibody. NB coupling into A-type JONs is unaffected, as were the ShakB plaques surrounding the GF dendrite. Scale bar in A: 20 μm for all panels.
(TIF)

S3 Fig Innexin immunostaining. Each figure is a maximum intensity projection of three 1μm confocal slices. The upper panels are from the antennal nerve (nerve) and the lower panels are taken from the region of neuropil around the GF dendrite (GF). On the left are immunostaining (red) and GFP (green) signals, on the right the immunostaining signal only in white. (A) ShakB immunoreactivity in control. Some patches of staining are present in the antennal nerve and large plaques are arrayed around the GF dendrite (arrow). (B) ShakB immunoreactivity in animal with RNAi knockdown of all isoforms (Vienna line). Only traces amounts of signal remain in nerve and neuropil. (C) ShakB immunoreactivity in animal with RNAi knockdown of all isoforms (Bloomington line). (D) Ogre immunoreactivity in a *peb*>GFP animal. Patches of staining are present in the antennal nerve, in axons and at the periphery. No Ogre staining is present around the GF dendrite. (E) Ogre immunoreactivity in a *JO15* > GFP animal. Patches of staining are present in the antennal nerve, in axons and at the periphery. No Ogre staining is present around the GF dendrite. (F) Ogre immunoreactivity in an animals with *JO15*>GFP and Ogre-RNAi. There is some reduction of staining compared to controls but immunoreactivity remains, although not associated with GFP labeling. (G) Inx2 immunoreactivity in a *peb*>GFP animal. Patches of staining are present in the antennal nerve, in axons and at the periphery. No Inx2 staining is present around the GF dendrite. (H) Inx7 immunoreactivity in a *peb*>GFP animal. Little specific staining is visible, in nerve or neuropil. For full genotypes see S1 Table. Scale bar in A: 20 μm for all panels.
(TIF)

S1 Table Genotypes tested. (XLSX)

S2 Table Putative off-targets of RNAi constructs. The RNAi constructs used in this study are listed, along with their sequences and putative off-targets, as determined by the software at FlyRNAi.org and the dscheck software at dscheck.rnai.jp. Note that the two methods predict different off-targets but that the short hairpin constructs are generally likely to be highly specific.
(XLSX)
