# Supplementary figures and images for: Shaking B Mediates Synaptic Coupling between Auditory Sensory Neurons and the Giant Fiber of Drosophila melanogaster
Source: PLoS One. 2016 Apr 4;11(4):e0152211. doi: 10.1371/journal.pone.0152211 (PMC4833477; doi:10.1371/journal.pone.0152211)

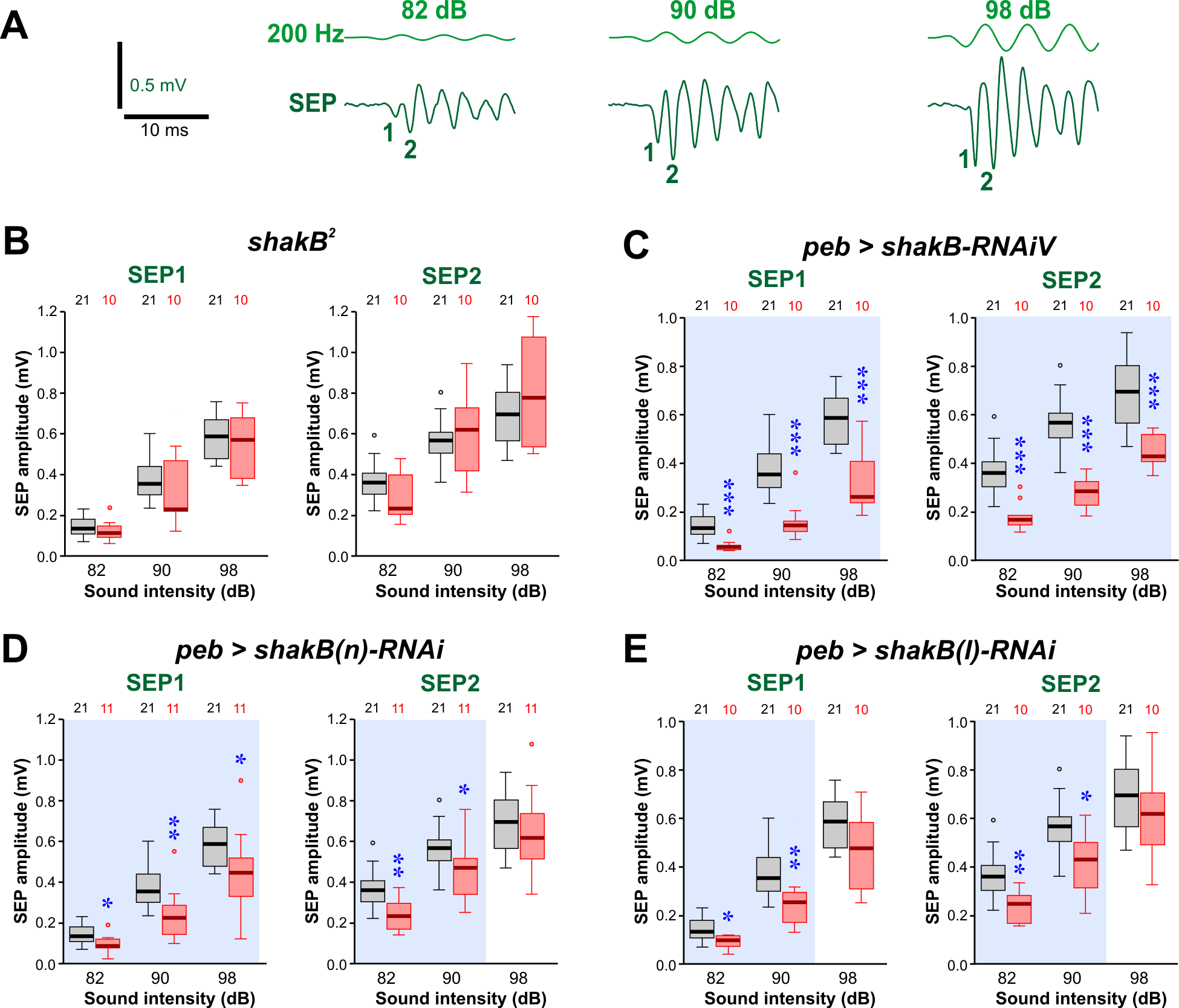

Supplement: S1 Fig — (A) Sample traces of sound-evoked potentials (SEPs) recorded from the base of the antenna, in response to 200 Hz sound pulses. (B) There is no significant difference in amplitudes of SEP1 and SEP2 in shakB2 mutants compared to controls (peb-GAL4 > Dcr-2). (C-E) Amplitudes of SEP1 and SEP2 in animals with different RNA interference constructs targeting shakB, compared to controls in which the RNAi was omitted. Paired t-tests or Mann-Whitney tests (with a Bonferroni correction for 3 comparisons) were used to determine significant differences from control. These differences are indicated with blue asterisks and a light blue background tint. (C) peb-GAL4 driving shakB-RNAi GD12666 (Vienna line, long hairpin) and UAS-Dcr-2. Both SEPs are reduced by about half. (D) peb-GAL4 driving shakB-RNAi JF02603 (targeted against shakB(n), long hairpin) and UAS-Dcr-2. Both SEPs were reduced in amplitude. (E) peb-GAL4 driving shakB-RNAi JF02604 (targeted against shakB(l), long hairpin) and UAS-Dcr-2. Both SEPs were reduced in amplitude. For full genotypes see S1 Table. (TIF) [file pone.0152211.s001.tif]

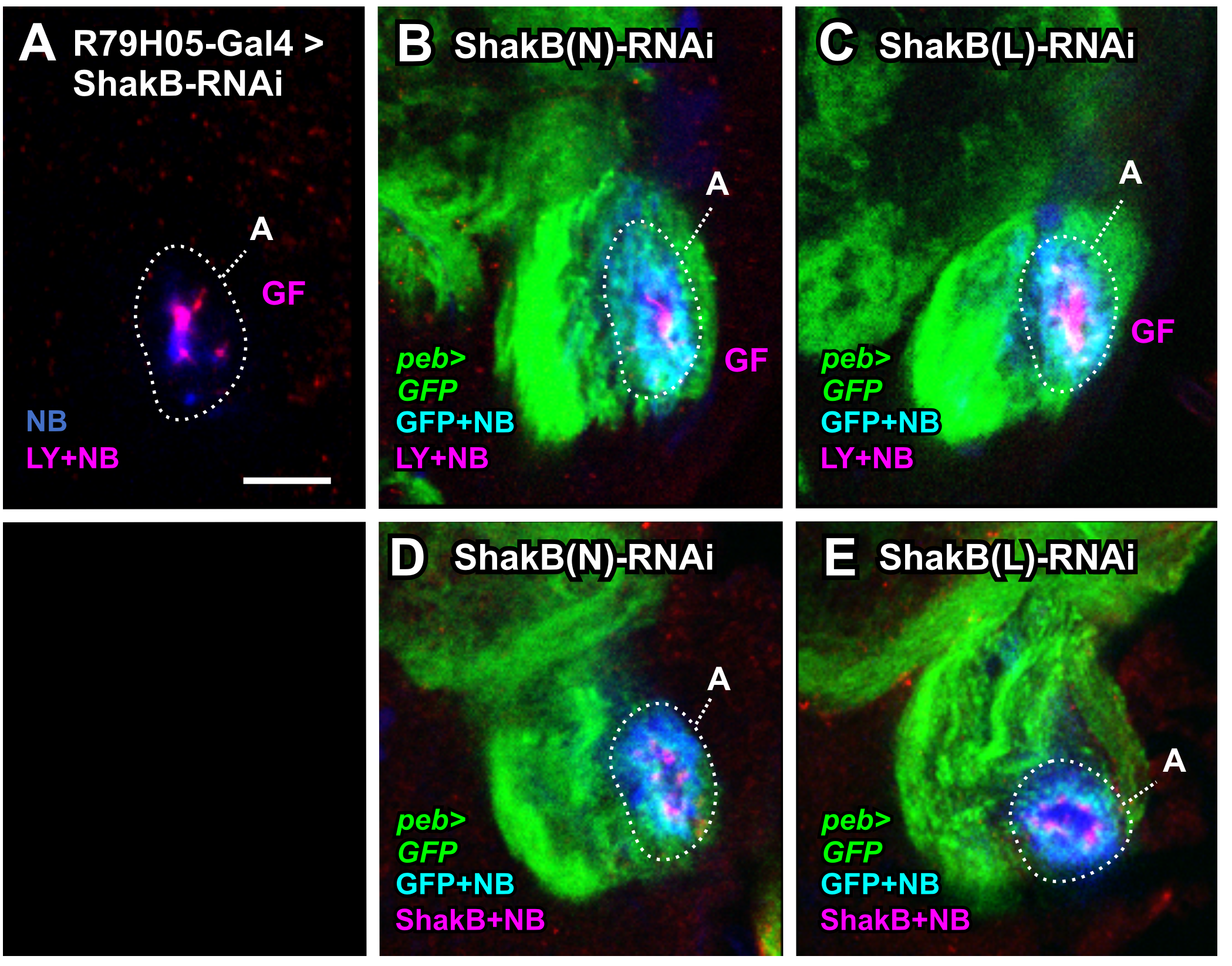

Supplement: S2 Fig — Each figure is a single frontal confocal slice. (A) ShakB-RNAi was driven by the R79H05-GAL4 driver in the GF. Dye coupling into A axons is prevented. (B-E) In these animals, peb-GAL4 was used to drive expression of CD8::GFP, and also Dcr-2. (B) ShakB(N) RNAi with JF02603. LY and NB were injected into the GF. NB coupling into A-type JONs is unaffected. (C) ShakB(L) RNAi with JF02604. LY and NB were injected into the GF. NB coupling into A-type JONs is unaffected. (D) ShakB(N) RNAi with JF02603. NB was injected into the GF and the preparation was stained with ShakB antibody. NB coupling into A-type JONs is unaffected, as were the ShakB plaques surrounding the GF dendrite. (E) ShakB(L) RNAi with JF02604. NB was injected into the GF and the preparation was stained with ShakB antibody. NB coupling into A-type JONs is unaffected, as were the ShakB plaques surrounding the GF dendrite. Scale bar in A: 20 μm for all panels. (TIF) [file pone.0152211.s002.tif]

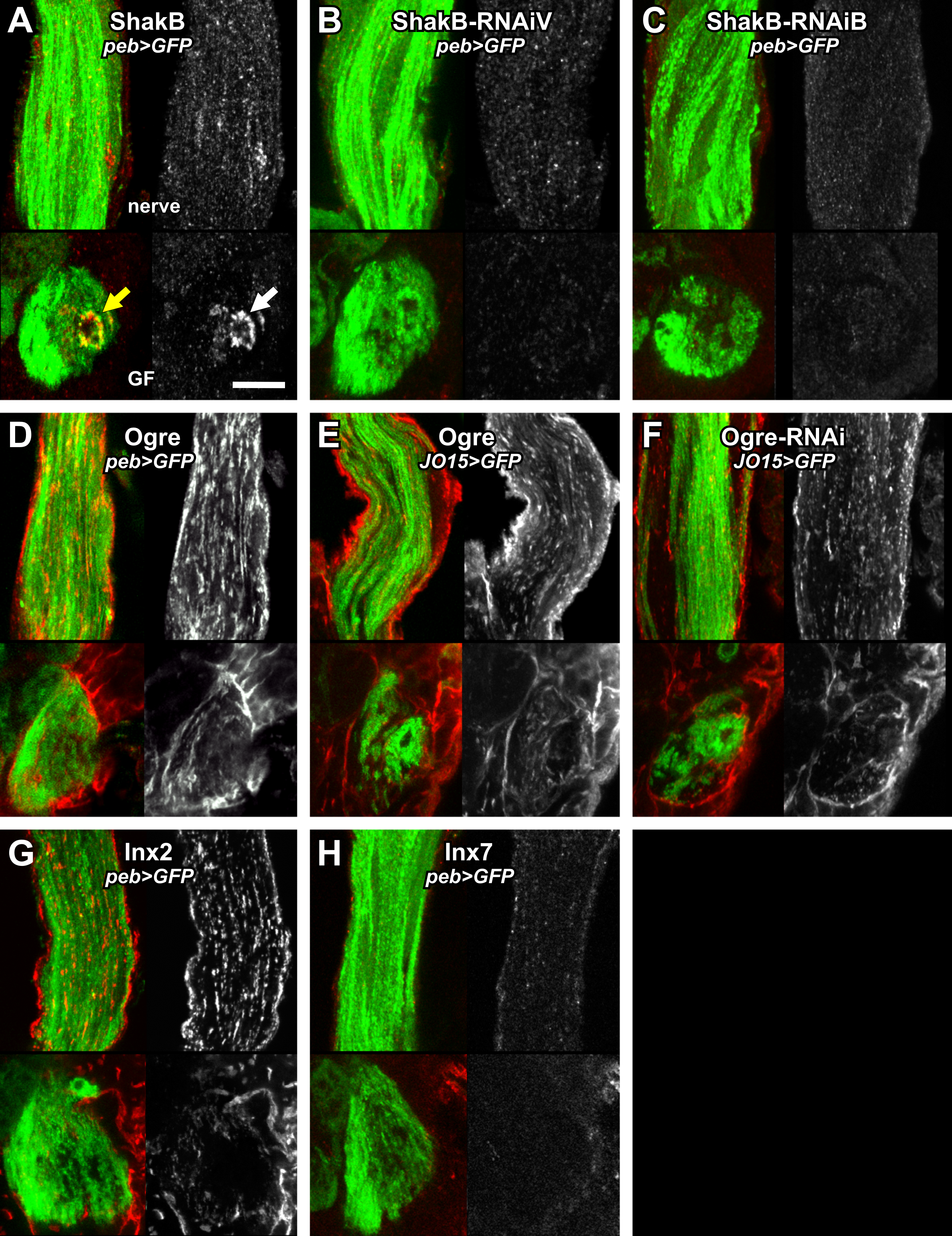

Supplement: S3 Fig — Each figure is a maximum intensity projection of three 1μm confocal slices. The upper panels are from the antennal nerve (nerve) and the lower panels are taken from the region of neuropil around the GF dendrite (GF). On the left are immunostaining (red) and GFP (green) signals, on the right the immunostaining signal only in white. (A) ShakB immunoreactivity in control. Some patches of staining are present in the antennal nerve and large plaques are arrayed around the GF dendrite (arrow). (B) ShakB immunoreactivity in animal with RNAi knockdown of all isoforms (Vienna line). Only traces amounts of signal remain in nerve and neuropil. (C) ShakB immunoreactivity in animal with RNAi knockdown of all isoforms (Bloomington line). (D) Ogre immunoreactivity in a peb>GFP animal. Patches of staining are present in the antennal nerve, in axons and at the periphery. No Ogre staining is present around the GF dendrite. (E) Ogre immunoreactivity in a JO15 > GFP animal. Patches of staining are present in the antennal nerve, in axons and at the periphery. No Ogre staining is present around the GF dendrite. (F) Ogre immunoreactivity in an animals with JO15>GFP and Ogre-RNAi. There is some reduction of staining compared to controls but immunoreactivity remains, although not associated with GFP labeling. (G) Inx2 immunoreactivity in a peb>GFP animal. Patches of staining are present in the antennal nerve, in axons and at the periphery. No Inx2 staining is present around the GF dendrite. (H) Inx7 immunoreactivity in a peb>GFP animal. Little specific staining is visible, in nerve or neuropil. For full genotypes see S1 Table. Scale bar in A: 20 μm for all panels. (TIF) [file pone.0152211.s003.tif]
